# Differential Peripheral Proteomic Biosignature of Fluoxetine Response in a Mouse Model of Anxiety/Depression

**DOI:** 10.3389/fncel.2017.00237

**Published:** 2017-08-16

**Authors:** Indira Mendez-David, Céline Boursier, Valérie Domergue, Romain Colle, Bruno Falissard, Emmanuelle Corruble, Alain M. Gardier, Jean-Philippe Guilloux, Denis J. David

**Affiliations:** ^1^CESP/UMR-S 1178, Université Paris-Sud, INSERM, Université Paris-Saclay Châtenay-Malabry, France; ^2^Proteomic Facility, Institut Paris Saclay d’Innovation Thérapeutique (UMS IPSIT), Université Paris-Sud, Université Paris-Saclay Châtenay-Malabry, France; ^3^Animal Facility, Institut Paris Saclay d’Innovation Thérapeutique (UMS IPSIT), Université Paris-Sud, Université Paris-Saclay Châtenay-Malabry, France; ^4^CESP/UMR 1178, Service de Psychiatrie, Faculté de Médecine, Université Paris-Sud, INSERM, Université Paris-Saclay, Hôpital Bicêtre Le Kremlin Bicêtre, France

**Keywords:** peripheral blood mononuclear cell, anxiety/depression mice, fluoxetine, biomarker, responder, major depressive disorders, major depressive episode, antidepressant

## Abstract

The incorporation of peripheral biomarkers in the treatment of major depressive disorders (MDD) could improve the efficiency of treatments and increase remission rate. Peripheral blood mononuclear cells (PBMCs) represent an attractive biological substrate allowing the identification of a drug response signature. Using a proteomic approach with high-resolution mass spectrometry, the present study aimed to identify a biosignature of antidepressant response (fluoxetine, a Selective Serotonin Reuptake Inhibitor) in PBMCs in a mouse model of anxiety/depression. Following determination of an emotionality score, using complementary behavioral analysis of anxiety/depression across three different tests (Elevated Plus Maze, Novelty Suppressed Feeding, Splash Test), we showed that a 4-week corticosterone treatment (35 μg/ml, CORT model) in C57BL/6NTac male mice induced an anxiety/depressive-like behavior. Then, chronic fluoxetine treatment (18 mg/kg/day for 28 days in the drinking water) reduced corticosterone-induced increase in emotional behavior. However, among 46 fluoxetine-treated mice, only 30 of them presented a 50% decrease in emotionality score, defining fluoxetine responders (CORT/Flx-R). To determine a peripheral biological signature of fluoxetine response, proteomic analysis was performed from PBMCs isolated from the “most” affected corticosterone/vehicle (CORT/V), corticosterone/fluoxetine responders and non-responders (CORT/Flx-NR) animals. In comparison to CORT/V, a total of 263 proteins were differently expressed after fluoxetine exposure. Expression profile of these proteins showed a strong similarity between CORT/Flx-R and CORT/Flx-NR (R = 0.827, *p* < 1e^-7^). Direct comparison of CORT/Flx-R and CORT/Flx-NR groups revealed 100 differently expressed proteins, representing a combination of markers associated either with the maintenance of animals in a refractory state, or associated with behavioral improvement. Finally, 19 proteins showed a differential direction of expression between CORT/Flx-R and CORT/Flx-NR that drove them away from the CORT-treated profile. Among them, eight upregulated proteins (RPN2, HSPA9, NPTN, AP2B1, UQCRC2, RACK-1, TOLLIP) and one downregulated protein, TLN2, were previously associated with MDD or antidepressant drug response in the literature. Future preclinical studies will be required to validate whether proteomic changes observed in PBMCs from CORT/Flx-R mice mirror biological changes in brain tissues.

## Introduction

Major Depressive Disorders (MDD) are the most frequent mental disorders worldwide (15% lifetime prevalence, 5% 1-year prevalence). MDD can lead to significant mortality, morbidity, reductions in quality of life, and have considerable costs implications to society (American Psychiatric Association, 2013). In addition, the main risk with MDD is suicide-related mortality. About 6–20% of patients suffering from MDD die by suicide (Isometsa, 2014). Selective Serotonin and/or Norepinephrine Reuptake Inhibitors are the most commonly prescribed drugs for the treatment of MDD. Despite recent advances in the pharmacological treatment of MDD, antidepressant drugs are only partially effective, with a 47% response rate and a 30% remission rate after the first-line treatment (Trivedi et al., 2006; Scarr et al., 2015). As most patients fail to enter remission with the first treatment, the incorporation of peripheral biomarkers in the treatment of MDD could supplement clinical observation and increase the remission rate (Breitenstein et al., 2014; Domenici, 2014; Chan et al., 2016a; Lam et al., 2016).

The study of proteins as potential disease or treatment biomarkers in the field of psychiatry, seems to be a straightforward approach since they are the main component of the cells and also drug targets (Breitenstein et al., 2014). Peripheral blood mononuclear cells (PBMCs) are circulating homogenous cells that can be easily collected and monitored across time in various species including humans and rodents. PBMCs may have a greater diagnostic power than a whole blood signature (Dhabhar, 2006). Interestingly, parallel transcriptomic changes have been observed using microarray in PBMCs and the brain, including the hippocampus in stressed mice (van Heerden et al., 2009). Yet, no biomarker has proven sufficient validity to be translated to the clinic (Leuchter et al., 2010). However, a few candidates associated with antidepressant response have recently emerged. For instance, similar change in protein expression in PBMCs (β-arrestin 1, a key regulator and scaffolds for G-protein-coupled receptor) has been observed between depressed treated-patients (Avissar et al., 2004) and depressed-like treated mice (Mendez-David et al., 2013, 2015). Recently, Svenningsson et al. (2014) showed that reduction in p11 in PBMCs could potentially predict antidepressant response to citalopram. Additionally, there is growing evidence of multiple dysregulated contributing factors, including growth factors, altered endocrine factors (Schmidt et al., 2011) or immune-related pathways (Belzeaux et al., 2016; Chan et al., 2016b) in mood disorders and/or in antidepressant responses. Thus, a viable alternative to the single-biomarker approach could be the development of biomarker panels to provide coverage of multiple biological factors that contribute to the heterogeneity of MDD and treatment response (Schmidt et al., 2011).

From “omic” approaches to brain imaging, different strategies may help to identify putative biomarkers of the pathophysiology and antidepressant response. Stimulated by the disappointing results of the genome wide association studies for antidepressant response, the potential of gene expression and proteomics as sources of predictive biosignatures have been explored (Labermaier et al., 2013). Applications of peripheral (PBMC, plasma, serum) proteomics, with the highest potential for having an impact on clinical practice, could be the identification of signatures or biomarkers which may predict antidepressant responses (Leuchter et al., 2010; Domenici, 2014).

Using a proteomic approach with high-resolution mass spectrometry technique, this study aimed to identify an indicative biosignature of fluoxetine response, which is commonly used as an antidepressant medication in PBMCs, and its isolated from a well-validated mouse model of anxiety/depression based on elevation of blood levels of glucocorticoids (David et al., 2009). We proposed that an overall estimation of mouse behavior based on response across complementary tests of anxiety/depressive-like behavior would be able to discriminate fluoxetine responders from non-responders. We hypothesized that a peripheral proteomic signature in isolated PBMCs would help in understanding the common and distinct effects of fluoxetine responders compared to non-responders.

## Experimental Procedures

### Subjects

Adult C57BL/6NTac male mice were purchased from Taconic Farms (Lille Skensved, Denmark). All mice were 7–8 weeks old, weighed 23–25g at the beginning of the treatment and were maintained on a 12L:12 D schedule (lights on at 0600). They were housed in groups of five. Food and water were provided *ad libitum*. The protocols involving animals and their care were conducted in conformity with the institutional guidelines that are in compliance with national and international laws and policies (Council directive #87-848, October 19, 1987, Ministère de l’Agriculture et de la Forêt, Service Vétérinaire de la Santé et de la Protection Animale, permissions # 92-256B to DJD) and in compliance with protocols approved by the Institutional Animal Care and Use Committee (CEE26 authorization #4747).

### Drugs

Corticosterone (4-pregnen-11b-DIOL-3 20-DIONE 21-hemisuccinate from Sigma (Sigma–Aldrich Saint-Quentin Fallavier, France) was dissolved in vehicle (0.45% hydroxypropyl-β-cyclodextrin, Sigma–Aldrich Saint-Quentin Fallavier, France). Fluoxetine hydrochloride (18 mg/kg per day in the drinking water) was purchased from Anawa Trading (Zurich, Switzerland).

### Corticosterone Model and Treatment

**FIGURE 1:**
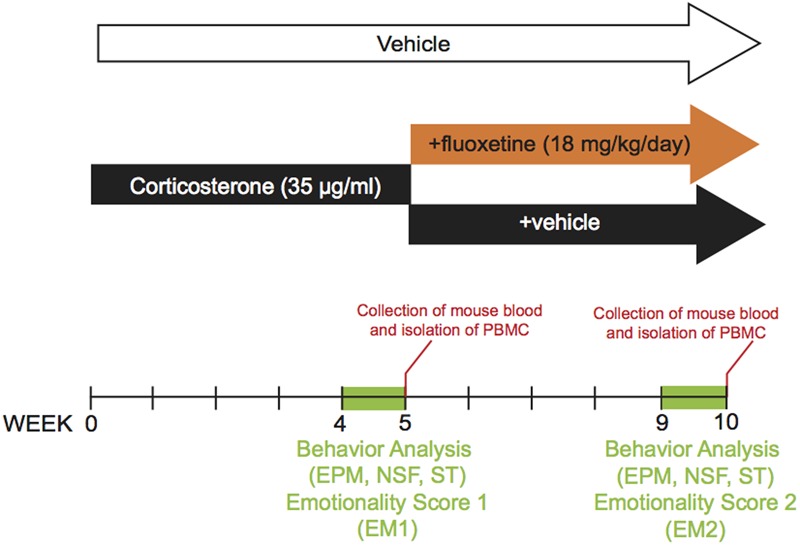
Timeline of experiments. In place of normal drinking water, grouped-housed male C57BL/6Ntac mice were presented during 10 weeks with vehicle (0.45% hydroxypropyl-β-cyclodextrin) or corticosterone (35 μg/ml) in the presence or absence of an antidepressant (fluoxetine, 18 mg/kg/day) during the last five weeks of the corticosterone regimen. Emotionality *z*-score was calculated after each behavioral session. Then, we investigated whether the behavioral changes induced after chronic corticosterone (week 4 to 5) were corrected by antidepressant treatment (week 9 to 10). The same animal was successively tested in the Elevated Plus Maze (EPM), the Novelty Suppressed Feeding (NSF), the Splash Test (ST) during both behavioral sessions. Peripheral Blood Mononuclear Cells were isolated from whole blood after each behavioral session.

The dose and duration of corticosterone treatment were selected based on previous study (CORT model, David et al., 2009; Mendez-David et al., 2013, 2014). Corticosterone (35 μg/ml, equivalent to about 5 mg/kg/day) or vehicle (0.45% β-cyclodextrine, β-CD) were available *ad libitum* in the drinking water in opaque bottles to protect it from light. Corticosterone-treated water was changed every 3 days to prevent any possible degradation. During the last 4 weeks of the protocol, corticosterone was delivered alone (*n* = 12 animals, CORT/V) or in the presence of fluoxetine (18 mg/kg/day, *n* = 46 animals, CORT/Flx) (see the experimental protocol on **Figure 1**). Treatments were maintained until the end of the experiments. Behavioral sessions to assess anxiety/depression-like phenotype and also the antidepressant response to fluoxetine occurred on week 4 and 9, respectively. Control animals received vehicle (vehicle/vehicle, VEH/V).

### Behavioral Experiment

#### Elevated Plus Maze (EPM)

The elevated plus maze (EPM) is a widely used behavioral assay for rodents and it has been validated to assess the anti-anxiety effects of pharmacological agents (for review Walf and Frye, 2007). This test was performed as described previously (Mendez-David et al., 2014). The maze is a plus-cross-shaped apparatus, with two open arms and two arms closed by walls linked by a central platform 50 cm above the floor. Mice were individually put in the center of the maze facing an open arm and were allowed to explore the maze for a duration of 5 min. The time spent in the maze and the numbers of entries into the open arms were used as an anxiety index. All parameters were measured using a videotracker (EPM3C, Bioseb, Vitrolles, France).

#### Novelty Suppressed Feeding (NSF)

The NSF is a conflict test that elicits competing motivations: the drive to eat and the fear of venturing into the center of a brightly lit arena. The latency to begin eating is used as an index of anxiety/depression-like behavior, because classical anxiolytic drugs as well as chronic antidepressants decrease this measure. The NSF test was carried out during a 10 min period as previously described (David et al., 2009). Briefly, the testing apparatus consisted of a plastic box (50 cm ×40 cm × 20 cm) the floor of which was covered with approximately 2 cm of wooden bedding. Twenty-four hours prior to behavioral testing, all food was removed from the home cage. At the time of testing, a single pellet of food (regular chow) was placed on a white paper platform positioned in the center of the box. Each animal was placed in a corner of the box, and a stopwatch was immediately started. The latency to eat (defined as the mouse sitting on its haunches and biting the pellet with the use of forepaws) was timed. Immediately afterwards, the animal was transferred to its home cage, and the amount of food consumed by the mouse in the subsequent 5 min was measured serving as a control for change in appetite as a possible confounding factor.

#### Splash Test (ST)

This test consisted of squirting a 10% sucrose solution on the mouse’s snout. This procedure induces grooming behaviors, due to the viscosity and palatability of the sucrose. The grooming behavior is sensitive to chronic stress or chronic corticosterone exposure and antidepressant treatment (Mendez-David et al., 2014). The total time spent in different grooming behaviors (i.e., face, paws, hindquarter, and shoulders) was directly recorded for 5 min in the home cage of the animals.

#### Behavioral Emotionality Measurement

Three behavioral tests (i.e., EPM, NSF, and ST) were used to measure components of animal behavioral emotionality. Z-score methodology was used to investigate the potential of combining results within and across the different behavior tests for depressive/ anxious-like behaviors and investigate the treatment effects in the CORT model. The emotionality-related data was normalized as previously described (Guilloux et al., 2011; Mekiri et al., 2017). Briefly, *z* scores are standardized scores (by the group mean and group standard deviation). They indicate how many standard deviations (σ) an observation (*x*) is above or below the mean of a control group (μ).

$$\text{Z}\;\text{=}\;\frac{\text{X-}\mu }{\sigma }$$

*Z* scores for behavioral measures were first averaged within the test and then across the test for equal weighting of the three tests comprising the final emotionality score. The increased behavioral emotionality was defined as decreased activity in the open arms in the EPM, increased NSF latency and decreased grooming in the splash test compared with control group means. The vehicle group was defined as the control. Thus, the emotionality score is not based on a single consistent behavior, but rather by a set of converging behavioral observations that together define an anxiety/depression-like phenotype. Emotionality score was calculated after the first and the second behavioral round. As we were interested in observing the differential antidepressant-response behaviors to global protein expression system level responses, behaviorally ambiguous mice were not used for mass spectrometry analysis described below.

### Isolation of Mouse Peripheral Blood Mononuclear Cells

To determine a biological signature of fluoxetine responders, the “most” affected animals of each group were used for proteomic analysis (5 for corticosterone/Vehicle, CORT/V; 7 for Corticosterone/fluoxetine responders, CORT/Flx-R; 6 for Corticosterone/fluoxetine non-responders, CORT/Flx-NR). The procedure was performed on unaenesthetized mice as previously described (Mendez-David et al., 2013). In compliance with the laboratory animal care guidelines, about 0.4 ml of blood per mice was collected in K_3_EDTA tubes using the submandibular bleeding method. The punctures were performed with 5 mm point size sterile lancets (MediPoint, Mineola, NY, United States) where the orbital vein and the submandibular vein join to form the jugular vein (Joslin, 2009). A light pressure with dry gauze was applied to the punctured area for hemostasis. Separation and extractions of PBMCs were done using the iodixanol mixer technique (Ford and Rickwood, 1990). Separations of mouse PBMCs were purified of mouse whole blood through density centrifugation (1,000 rpm at 20°C for 30 mn) using solution B with the OptiPrep^TM^gradient solution (Sigma–Aldrich Saint-Quentin Fallavier, France). After centrifugation, OptiPrep^TM^gradient solution separated layers of blood, with PBMCs under a layer of plasma. The PBMCs layers were carefully removed from the tube and transferred to a new 50 mL conical tube and were washed twice with solution B. After centrifugations (1,200 rpm at 20°C for 7 mn) and several washing steps, mouse PBMCs were recovered with a last centrifugation (3,000 rpm at 4°C for 5 mn) and stored at -80°C before subsequent assay.

### Proteomics Analysis

#### Protein Separation

Protein extracts from PBMCs were homogenized in solution solubilization (Urea 7M, Thiourea 2M, CHAPS 3%, Nonidet P-40 1%, DTT 1%). Protein concentration was measured using 2D-Quant kit (GE Healthcare, France) and 15 μg of proteins were loaded and separated by 12 % SDS-PAGE. A short migration was then performed (7 min, 80 V, 25 W followed by 4 min, 200 V, 25 W) and gels were stained with Coomassie colloidal blue (EZblue, Sigma–Aldrich, France).

#### Protein In-Gel Digestion

Portions of gel that contain all proteins were cut and digested as followed: pieces of gel were successively washed and de-stained with water, acetonitrile (ACN) and 25 mM ammonium bicarbonate (NH_4_HCO_3_). A reduction/alkylation step was performed with dithiothreitol (DTT) 10 mM and iodoacetamide 55 mM. Gels were dehydrated with acetonitrile and rehydrated at 4°C in 12 ng/μL sequencing grade modified trypsin (Promega, France) solubilized in 25 mM NH_4_HCO_3_ in 1 h and then digested at 37°C overnight. After tryptic digestion, peptides were extracted by incubating gel pieces in extraction solvent (0.5% trifluoroacetic acid (TFA)/50% ACN) for 15 min and in ACN for 15 min at room temperature. Supernatants were vacuum dried. The dried extract peptides were dissolved in 50 μl of loading buffer (0.08% TFA/2% ACN) just before mass spectrometry analysis.

#### Mass Spectrometry Analysis

Four microliters of sample were loaded on the nano-UPLC Ultimate 3000 RSLCnano (Thermo). Sample was loaded at 20 μL/min on the pre-column cartridge (PepMap 100 C18, 5 μm; 300 μm i.d., 5 mm, Thermo Scientific) and peptides were then separated with a gradient of acetonitrile on the reverse phase column PepMap 100 C18 (stationary phase: C18, 3 μm; column: 75 μm i.d., 500 mm; nanoViper, Thermo Scientific, France). Buffers were 0.1% formic acid in 2% acetronitrile (A) and 0.1% formic acid in 80% acetonitrile (B). The peptide separation was realized during 64 min at 300 nL/min with a linear gradient from 0 to 45% B for 55 min followed by a gradient from 45 to 98% B for 5 min. Eluted peptides were analyzed on-line with a high-resolution mass spectrometer Orbitrap Fusion Lumos Tribrid (Thermo Scientific, France) using a nanoelectrospray interface in positive polarity mode, on PAPPSO platform^1^. Peptide ions were analyzed using Xcalibur 3.0 (Thermo Scientific, France) with following data-dependent acquisition steps: (1) full MS scan in orbitrap (mass-to-charge ratio [*m*/*z*] = 400–1500; mass tolerance, ±10 ppm) and (2) MS/MS in Ion Trap with CID activation (collision energy, 35%; activation time, 30 ms; centroid mode). Dynamic exclusion time was set to 60 s.

### Statistical Analysis

#### Behavioral Analysis

To assess the behavioral consequence of a chronic corticosterone treatment in the EPM, NSF, and ST or emotionality scores, Student’s *t*-tests were performed and results were expressed as mean ± SEM values. For the behavioral analysis after chronic fluoxetine treatment, a one-way ANOVAs was applied to the data as appropriate. Significant main effects were followed by Fisher’s *Post hoc* test. With regards to the NSF test, we used the Kaplan–Meier survival analysis owing to the lack of normal distribution of the data. Mantel–Cox log rank test was used to evaluate differences between experimental groups. Statistical significance was set at *P* < 0.05. Data were analyzed using Prism 6.0h software (GraphPad, La Jolla, CA, United States).

#### Data Processing and Bioinformatics Analysis

Peak lists were generated as mzXML files using the converter MSConvert (ProteoWizard). A database search was performed using X!TandemPipeline software developed by PAPPSO facility^2^ (version 3.4.3) (Langella et al., 2017) with search parameters as followed: enzymatic cleavage by trypsin digestion with one possible miscleavage; fixed carbamido-methylation modification on cysteine and variable oxidation on methionine; precursor mass tolerance of ±10 ppm and fragment mass tolerance of 0.5 Da. Several databases were used: the Uniprot KB/SwissProt Mus musculus database (24977 entries, version January 2017) and a homemade contaminant database (trypsin, keratine, etc.). The identified proteins were filtered with a minimum of two different peptides required with a peptide *E-value* < 0.01, and a protein *E-value* (product of unique peptide *E values*) < 10^-4^. Combine analysis mode with all samples was performed and results collected from grouping proteins: proteins, which have at least one peptide in common. This allowed to group proteins with similar functions. Within each group, proteins with at least one specific peptide relatively to other members of the group were reported as subgroups. One subgroup represents one specific protein. Proteins are characterized with their spectral number. Label free quantification of proteins were achieved with spectral counting approach (SC), which is a strategy to determine a relative quantification of protein from their number of spectra obtained with tryptic peptides in MS. This quantification is based on the fact that more of a particular protein is present in a sample; more MS spectra are detected for peptides of that protein. Statistical analysis was performed using MassChroqR package developed by PAPPSO team^3^ (R version 3.3.2). A generalized linear mixed model (GLM) with a Poisson distribution was applied. This model suits in the case of a counting like SC. The principal component analysis was obtained by simulating the kernel densities from group’s means and variances assuming bivariate normal distributions. This distribution was generated using protein abundances as variables. Hierarchical bivariate clustering was performed using Euclidean distances and unweighted pair group averages as the aggregation method. All data analyses and graphical representations were performed using the R package MassChroq. Significant changes in protein abundance were determined by analysis of variance (ANOVA) using a Chi-square test. Treatment effect was considered with an adjusted *p*-value for multiple testing by a Benjamini–Hochberg procedure (Benjamini and Hochberg, 1995). Student’s *t*-tests were performed to identify proteins with significant differences expressed between groups with the following criteria: *p*-value was set at < 0.05.

#### Functional Analysis

Selected proteins were overlaid on the global molecular network of Ingenuity Pathway Analysis^4^ (Ingenuity^®^Systems) allowing for a generation of gene networks based on their connectivity. Their score takes into account the relative numbers of network eligible molecules, of molecules analyzed and the total number of molecules in Ingenuity’s knowledge base. IPA generates disease links on the literature-based association with illness.

## Results

Detailed statistical results for behavior are provided in **Supplementary Table S1**.

### A 4-week Corticosterone Treatment Induced Anxiety/Depression-Like Phenotype

**FIGURE 2:**
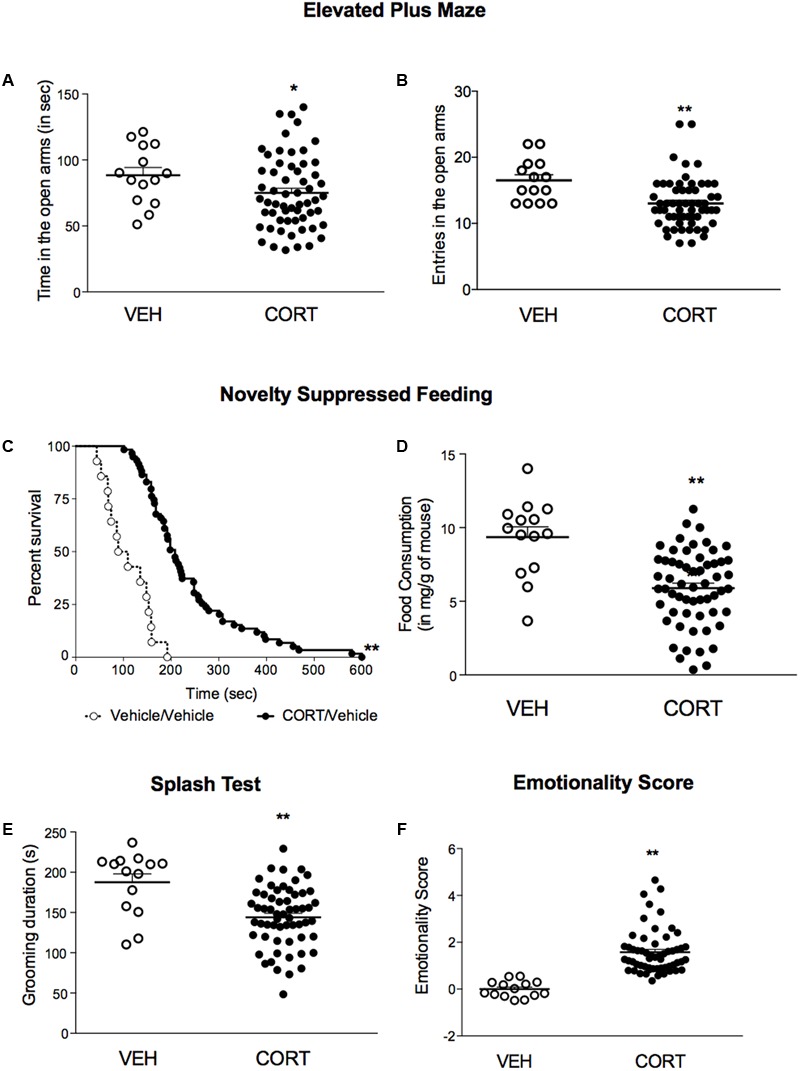
A 4-week corticosterone treatment (35 μg/ml) induced an anxiety/depression-like phenotype in C57BL/6Ntac mice. **(A,B)** Effects of corticosterone (35 μg/ml, CORT) regimen on anxiety behaviors in the Elevated Plus Maze (EPM). Anxiety, measured for various parameters is expressed as mean total time in seconds **(A)** or entries **(B)** in open arms of EPM paradigm. **(C)** Effects of 4 weeks of corticosterone regimen (35 μg/ml) on anxiety- and depression related behaviors in the Novelty Suppressed Feeding paradigm. Results are expressed as cumulative survival with percentage of animals that have not eaten over 10-min. **(D)** Effects of 4 weeks of corticosterone regimen (35 μg/ml, CORT) on depression related behaviors in the Splash Test (ST). Results are expressed as mean of grooming duration (in seconds). **(E)** Effects of 4 weeks of corticosterone regimen (35 μg/ml, CORT) on food consumption in the Novelty Suppressed Feeding paradigm. Results are expressed as mean of food consumption (in mg/g of mouse). **(F)** Effects of 4 weeks of corticosterone regimen (35 μg/ml, CORT) on anxiety/depression-like behaviors on the emotionality score. Test *Z*-values (elevated plus maze, novelty-suppressed feeding and splash test) are calculated by averaging individual *Z*-scores to obtain emotionality *Z*-scores. Values plotted are mean ± SEM [*n* = 14 and 59 animals for vehicle (VEH, open circle) and corticosterone (CORT, black dot) per group respectively]. Unpaired *t*-test (^∗^*p* < 0.05, ^∗∗^*p* < 0.01 *versus* VEH group) or Kaplan–Meier survival analysis followed by Mantel–Cox log-rank test were applied (^∗∗^*p* < 0.01 *versus* VEH group).

Using a low dose of corticosterone (35 μg/ml) for 4 weeks, we demonstrated that C57BL/6Ntac treated mice developed an anxiety/depression-like phenotype in the EPM, NSF and Spash tests (**Figure 2**) as previously described (David et al., 2009; Mendez-David et al., 2014). Indeed, a decrease in time spent and in entries in the open arms (**Figures 2A,B**, *p* < 0.05, and *p* < 0.01, respectively), an increase in the latency to feed (**Figures 2C,D**, *p* < 0.01) and a decrease in grooming duration (**Figure 2E**, *p* < 0.01) were observed in corticosterone-treated mice. *Z*-score normalization was then performed, within the respective behavioral parameters, hence transforming absolute values to numbers of standard deviations from the vehicle means. *Z*-scoring across complementary behavioral dimensions provided a more robust overall assessment of the effect of stress on behavioral emotionality (Guilloux et al., 2011). Thus, chronic corticosterone treatment induced an anxiety/depressive-like phenotype in mice, as measured by an increase in the emotionality score (**Figure 2F**, *p* < 0.01).

### Effects of a 4-week Treatment with Fluoxetine in a Stress-Related Model of Anxiety/Depression

**FIGURE 3:**
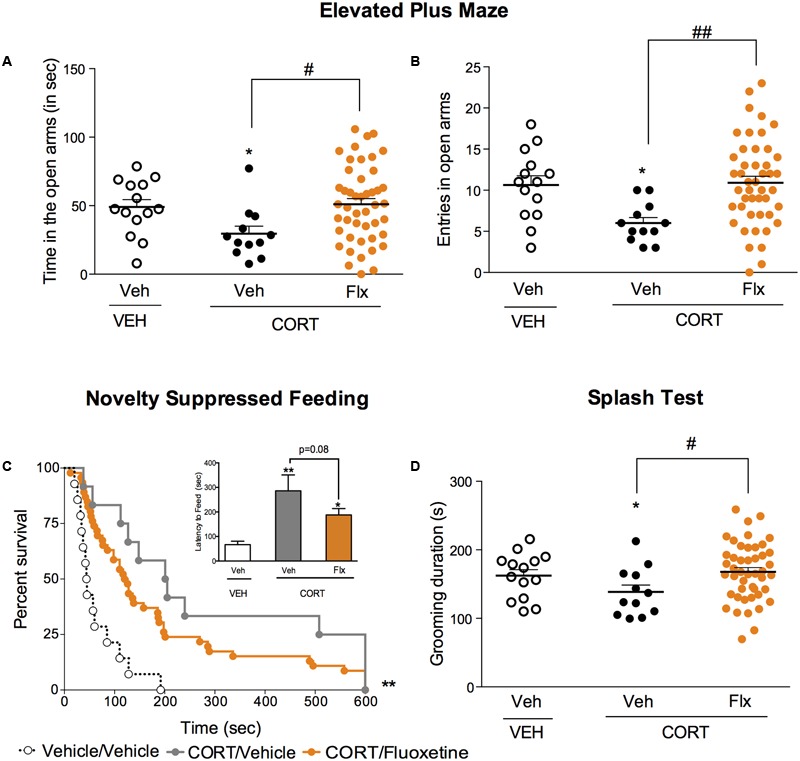
Chronic fluoxetine treatment produces anxiolytic-like and antidepressant-like effects in a mouse model of anxiety/depression. **(A,B)** Effects of 4 weeks of fluoxetine treatment (18 mg/kg/day, Flx) on anxiety behaviors in the Elevated Plus Maze (EPM). Anxiety, measured for various parameters is expressed as mean total time in seconds **(A)** or entries **(B)** in open arms of EPM paradigm. **(C)** Effects of 4 weeks of fluoxetine treatment (18 mg/kg/day, Flx) on anxiety- and depression related behaviors in the Novelty Suppressed Feeding paradigm. Results are expressed as cumulative survival with percentage of animals that have not eaten over 10-min or mean of latency to feed (in seconds) (inset). **(D)** Effects of 4 weeks of fluoxetine treatment (18 mg/kg/day, Flx) on depression related behaviors in the Splash Test (ST). Results are expressed as mean of grooming duration (in seconds). Values plotted are mean ± SEM [*n* = 14, 12, and 46 animals for vehicle/vehicle (VEH/V, open circle) corticosterone/vehicle (CORT/V, black dot) and corticosterone/fluoxetine (CORT/Flx, orange dot) per group respectively]. One-way ANOVA Fisher’s PLSD post hoc analysis (^∗^*p* < 0.05, ^∗∗^*p* < 0.01 *versus* Veh/V group; ^#^*p* < 0.05, ^##^*p* < 0.01 *versus* CORT/V group) or Kaplan–Meier survival analysis followed by Mantel–Cox log-rank test were applied (^∗∗^*p* < 0.01 *versus* Veh/V group).

We then explored whether a chronic fluoxetine treatment was able to correct the anxiety/depressive-like state induced by chronic corticosterone. In the EPM, a chronic treatment with fluoxetine corrected corticosterone-induced decrease in time and entries in the center (**Figures 3A,B**, *p* < 0.05, and *p* < 0.01, respectively). In the NSF, a trend for a reduction of chronic corticosterone-induced increase in latency to feed was observed after chronic fluoxetine treatment (**Figure 3C**, Kaplan–Meier survival analysis, Mantel-Cox log-rank test, *p* < 0.01, *p* = 0.08 for the insert bar chart). Finally, after squirting a 10% sucrose solution on the mouse’s snout, the decrease in grooming frequency observed in chronic corticosterone animals was reversed by chronic fluoxetine treatment (**Figure 3D**, *p* < 0.05).

### Responders and Non-Responders to Chronic Fluoxetine Treatment in Corticosterone-Induced Anxiety/Depression-Like Phenotype

Chronic corticosterone-treated animals were distributed for the last 4 weeks of treatment in a way that no significant difference occurred between the two groups (CORT-V and CORT/Flx) (**Supplementary Figure S1A**). At the end of the study, we also ensured that CORT/Flx-R and CORT/Flx-NR did not differ in their emotionality score before fluoxetine treatment.

**FIGURE 4:**
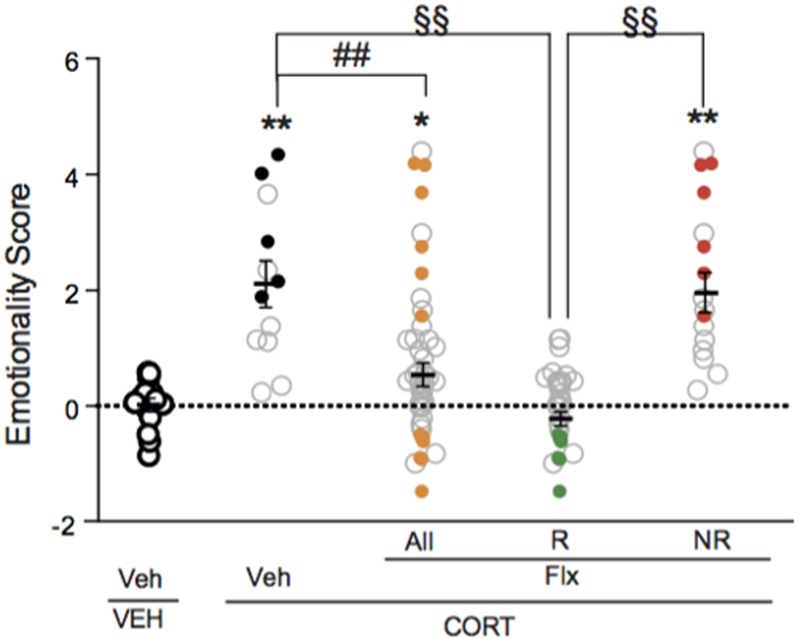
Two thirds of chronic fluoxetine-treated animals block stress-induced increase in emotionality *z*-score. Normalization of data using *z*-score method was performed for each behavioral parameter in EPM, NSF and ST after the second session of behavior. Test *z*-values were then calculated by averaging individual *z*-scores, and averaged to obtain the emotionality score. Values plotted are mean ± SEM [*n* = 14, 12, 30, and 16 animals for vehicle/vehicle (VEH/V, open circle), corticosterone/vehicle (CORT/V, black dot), corticosterone/fluoxetine responder (CORT/Flx-R, green dot) and corticosterone/fluoxetine non-responder (CORT/Flx-NR, red dot) per group, respectively]. Colored dot (CORT/V, black dot; CORT/Flx-R, green dot; CORT/Flx-NR; red dot) are the “most” affected animals selected for the proteomic approach. One-way ANOVA Fisher’s PLSD *post hoc* analysis (^∗^*p* < 0.05, ^∗∗^*p* < 0.01 *versus* Veh/V group; ^##^*p* < 0.01 *versus* CORT/V group; ^§§^*p* < 0.01 *versus* CORT/Flx-R group).

A one-way ANOVA after the second behavioral session revealed a significant effect of fluoxetine on emotionality score. Applying z-normalization across tests after the second round of behavior demonstrated that chronic fluoxetine decreased CORT-induced anxiety/depression-like phenotype in mice, as measured by a decrease in emotionality score (**Figure 4**, *p* < 0.01 *versus* VEH/V group; *p* < 0.01 *versus* CORT/V group), confirming previous studies (Guilloux et al., 2011; Mendez-David et al., 2015).

Interestingly, in fluoxetine-treated mice, phenotypic variations were observed as two groups of mice could be distinguished according to their emotionality score (**Figure 4** and **Supplementary Figure S1A**). Clinical response to antidepressants in depression is defined as a 50% decrease in rating scale score (Nierenberg and DeCecco, 2001). Thus, we also analyzed the behavioral data according to the 50% cut-off. A fluoxetine-treated animal with a 50% decrease in emotionality score was defined as CORT/Flx-R, whereas a decrease in this score below 50%, indicates a CORT/Flx-NR animal. Of 46 fluoxetine-treated animals, 65.2% (30 mice) responded to fluoxetine with an average decrease of 1.74 points in emotionality score between both behavioral sessions (**Supplementary Figure S1B**, *p* < 0.01 *versus* VEH/V group; *p* < 0.01 *versus* CORT/V group; *p* < 0.01 *versus* corticosterone group). In contrast, in CORT/Flx-NR animals (16 mice out of 46), a 0.82 points increase in emotionality score was observed between the two rounds of behavior, pointing out the absence of anxiolytic/antidepressant-like effect of fluoxetine in these animals (**Supplementary Figure S1B**).

After the behavioral sessions and to determine a biological signature of fluoxetine response, PBMCs from the “most” affected animals of each group (5 for CORT/V, 7 for CORT/Flx-R and 6 for CORT/Flx-NR) were isolated for further proteomic analysis (**Supplementary Figure S2**).

### Common and Differential Proteomic Changes in Responders and Non-Responders to Fluoxetine

**FIGURE 5:**
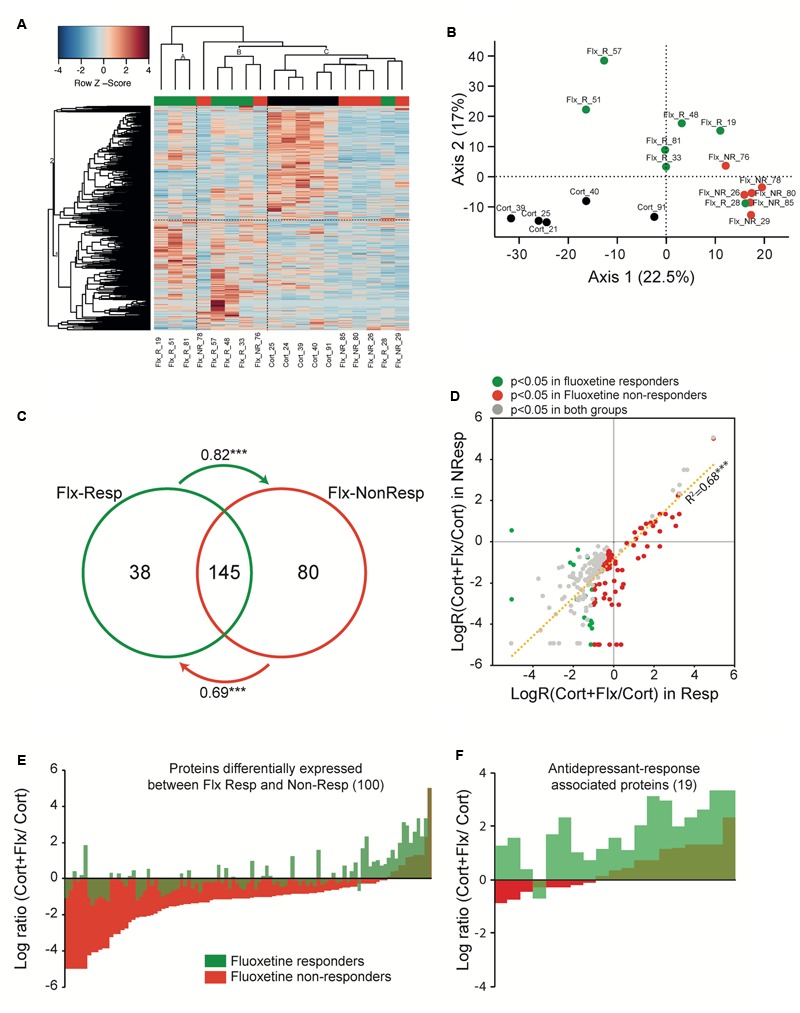
Peripheral proteomic changes after fluoxetine exposure in responders and non-responders. **(A)** Hierarchical bivariate clustering of expression profiles of animals (column) and proteins (rows) depicts the differences between CORT/V (black rectangle), CORT/Flx-R (green rectangle), and CORT/Flx-NR (red rectangle) groups. An animal’s expression is red for above-average values, and blue for below-average values. **(B)** Principal Component Analysis of expression profiles revealed 2 main axis separating results. **(C)** Venn diagram of protein levels with significant fluoxetine effect in CORT/Flx-R and CORT/Flx-NR groups. Changes affects massively common proteins between these two groups. Changes associated solely with CORT/Flx-R (38) or CORT/Flx-NR (80) strongly correlated between each other, as indicated by the arrows. Arrows indicate directional correlations between changes in protein expression for protein identified significant in one group (origin of arrow) and changes for the same protein in the other group (end of arrow). **(D)** Overall directional changes of protein expression were strongly correlated between groups. **(E)** Hundred proteins were observed significantly differentially expressed between CORT/Flx-R and CORT/Flx-NR groups, however **(F)** only 19 out of 100 could be associated with response to fluoxetine. ^∗∗∗^*p* < 0.0001 after Pearson χ^2^ correlation analysis.

Using a high resolution mass spectrometry analysis by X!Tandem Pipeline 1245 specific proteins (*n* = 5–7 samples per group) were detected in PBMCs (**Supplementary Tables S2, S3**). Characterized proteins with less than two peptides were excluded, leading to identification of 938 proteins. Hierarchical clustering of the expressed proteins distinguished CORT/V treated animals from CORT/Flx-R and CORT/Flx-NR, however, with some overlap (**Figure 5A**). This aggregate behavior of this large-scale systemic response was quantified with Principal Components Analysis (PCA, **Figure 5B**), which confirmed hierarchical clustering analysis. According to PCA, proteins’ abundance separated CORT/V, CORT/Flx-R and CORT/Flx-NR.

We observed a group effect for 305 proteins (*p* < 0.05, **Supplementary Table S4**). We found changes in expression levels of 183 proteins that were significantly altered by chronic fluoxetine in CORT/Flx-R mice, and 225 in CORT/Flx-NR (*p* < 0.05, **Figure 5C** and **Supplementary Table S4**). Among the proteins affected by fluoxetine, we observed a strong overlap across CORT/Flx-R and CORT/Flx-NR groups, with 145 proteins observed in common. Interestingly, protein changes observed solely in responders displayed a similar trend of expression in the CORT/Flx-NR group (arrow in **Figure 5C**, Pearson correlation value *R* = 0.82, *p* < 1e^-7^), and *vice-versa* (*R* = 0.69, *p* < 1e^-7^). These response-independent effects of fluoxetine treatment on PBMCs proteome were also reflected in **Figure 5D**. Indeed, 263 proteins were differentially affected by fluoxetine (compared to CORT) either in responders, non-responders or both with strong similarities of expression between responders and non-responders (*R* = 0.827, *p* < 1e^-7^).

Direct comparison of CORT/Flx-R and CORT/Flx-NR groups revealed 100 proteins differentially expressed between responders and non-responders (*p* < 0.05, **Figure 5E**). Among these proteins, 19 of them were associated with treatment response. Their expression was significantly different between CORT/Flx-R and CORT/Flx-NR, significantly different between CORT/Flx-R and CORT/V-treated animals and the direction of expression changes was either opposite or of greater amplitude in the CORT/Flx-R group compared to CORT/Flx-NR (**Figure 5F**). Interestingly, an IPA analysis revealed “*protein ubiquitination pathways*”, “*interleukin 1 signaling”* and “*metabolic diseases”* as the top canonical pathways diseases and biological functions associated with these 19 proteins, respectively (**Table 1**). Moreover, a literature search performed among these proteins using PubMed and Google Scholar indicated that 9 of them have been previously associated with antidepressant drug response or with MDD in clinical or preclinical studies (8 upregulated: RPN2, HSPA9, NPTN, AP2B1, UQCRC2, RACK-1, TOLLIP, and 1 downregulated protein, TLN2, **Table 2**).

**Table 1:** Ingenuity pathway analysis for functional analysis of the mapped biological functions and/or disease categories and canonical pathways for proteins in peripheral blood mononuclear cells.

Top canonical pathways	*p*-value
**Name**	
Protein Ubiquitination Pathway	1.46E-03
IL-1 Signaling	2.99E-03
Ketolysis	8.07E-03
Ketogenesis	8.96E-03
Mevalonate Pathway I	1.16E-02

**Top upstream regulators**	

**Upstream regulator**	
5-fluorouracil	4.71E-5
dichlorovinylcysteine	3.14E-04
ST1926	4.05E-04
CD 437	8.03E-04
CREB-NFkB	9.09E-04

**Top disease and bio functions**	

**Diseases and disorders**	
Metabolic Disease	2.14E-02–1.15E-05
Cancer	4.32E-02–1.60E-05
Hematological Disease	4.32E-02–1.60E-05
Immunological Disease	4.32E-02–1.60E-05
Organismal Injury and Abnormalities	4.32E-02–1.60E-05
**Molecular and cellular functions**	
Lipid Metabolism	3.45E-02–2.75E-05
Nucleic Acid Metabolism	1.16E-02–8.99E-05
Small Molecule Biochemistry	2.14E-02–8.99E-04
Cellular Movement	2.14E-02–8.99E-04
Cell Death and Survival	3.89E-02–8.99E-04

**Top Networks**	**Score**

**ID Associated Network Functions**	
1. Developmental Disorder, Hereditary Disorder, Metabolic disease	28
2. Behavior, Nervous System Development and Function, Cell-To-Cell Signaling and Interaction	19

*19 proteins showed significant differential direction of expression in fluoxetine responder and non-responder mice, e.g., overexpressed in one condition and under expressed in the other.*

**Table 2:** Identification of the 19 proteins showing significant differential direction of expression in fluoxetine responder (CORT/Flx-R) and fluoxetine non-responder (CORT/Flx-NR) mice.

Protein ID	Protein name	Regulation
Q8K021	SCAMP1_MOUSE Secretory carrier-associated membrane protein 1	Up
**Q9DBG6**	**RPN2_MOUSE Dolichyl-diphosphooligosaccharide-protein glycosyltransferase subunit 2**	
**P38647**	**HSPA9_MOUSE Heat shock protein 9**	
Q8VDM4	PSMD2_MOUSE 26S proteasome non-ATPase regulatory subunit 2	
**P97300-1**	**NPTN_MOUSE Isoform 1 of Neuroplastin**	
**O35643**	**AP1B1_MOUSE AP-1 complex subunit beta-1**	
Q8QZT1	ACAT1_MOUSE Acetyl-CoA acetyltransferase, mitochondrial	
Q8BWT1	Acaa2_MOUSE 3-ketoacyl-CoA thiolase, mitochondrial	
**Q9DBG3-2**	**AP2B1_MOUSE adaptor-related protein complex 2, beta 1 subunit**	
**Q9DB77**	**UQCRC2_MOUSE ubiquinol cytochrome c reductase core protein 2**	
**P68040**	**RACK1_MOUSE Receptor of activated protein C kinase 1**	
O08579	EMD_MOUSE Emerin	
Q8BY89-2	SLC44A2_MOUSE solute carrier family 44, member 2	
P12388	SERPINB2_MOUSE serine (or cysteine) peptidase inhibitor, clade B, member 2	
**Q9QZ06**	**TOLLIP_MOUSE Toll-interacting protein**	
O09061	PSMB1_MOUSE Proteasome subunit beta type-1	
P70670	NACA_MOUSE Nascent polypeptide-associated complex subunit alpha, muscle-specific form	
Q8VEK0	TMEM30A_MOUSE transmembrane protein 30A	
**Q71LX4**	**TLN2_MOUSE Talin-2**	**Down**

*A literature search was performed using PubMed and Google Scholar. We used the Clinical Queries search filters were “each identify protein” and “antidepressant response” or “major depressive disorder” and indicate in the positive match.*

## Discussion

Current antidepressant drug treatments are not sufficient, as many patients do not adequately respond. Animal models, such as the CORT model, are valuable in providing a translational framework to study SSRI insensitivity and to validate findings as potential biomarkers for treatment responsiveness in humans. This kind of approach will pave the way for novel approaches and therapeutic strategies for relieving depressive disorders. Here, using the mouse CORT-model associated with an emotionality score analysis and proteomic identification in PBMCs, we developed a novel approach to determine a protein expression profile between CORT/Flx-NR and CORT/Flx-R (Mendez-David et al., 2015).

### Behavioral Emotionality and PBMCs Isolation from Responders and Non-responders to Fluoxetine in a Mouse Model of Anxiety/Depression

Current animal models are classically used to test whether antidepressant drugs can reverse stress-induced anxiety/depression-like phenotype. In the literature, antidepressant response in these models is defined by comparing the mean results of the behavioral task across control and treated groups, without distinguished responders from non-responders (Allen et al., 2012). Applying this classic methodology, we confirmed that chronic corticosterone-induced anxiety/depression-like phenotype is overall corrected by chronic fluoxetine treatment (Mendez-David et al., 2013, 2014; Darcet et al., 2014, 2016). Additionally, we used scatterplots representations of the emotionality score to observe the distribution of the results and separate responders and non-responders to chronic antidepressant treatment. Mice that participated in the behavioral tests (EPM, NSF, ST) showed a different distribution in response to antidepressant treatment. Interestingly, previous observations in the NSF already showed that not all CORT-treated mice respond to chronic antidepressant treatment, thus resulting in a bimodal distribution (Samuels et al., 2011). Here, the selection of animals responding or not responding to chronic fluoxetine was not based on results obtained in a single test, which is classically observed (Christensen et al., 2011; Samuels et al., 2011; Bisgaard et al., 2012; Walker et al., 2015; Park et al., 2016). Rather, the use of the behavioral emotionality score covers the multiple aspects of emotional phenotype. Thus, fluoxetine-treated animals were subdivided in CORT/Flx-R and CORT/Flx-NR according to a 50% decrease in emotionality score cut off as defined in clinic (Nierenberg and DeCecco, 2001). Our study revealed a fluoxetine-responding rate of 65%, which is similar to what is observed in clinic. Using this approach augmented the translational validity of the CORT model to define a biological signature of antidepressant response.

Previously, we showed that PBMCs isolated from a small volume of whole blood in unanesthetized mice using a submandibular bleeding method provided a useful biological tool that assess circulating protein expressions and allowed the screening of potential biomarkers for antidepressant response (Mendez-David et al., 2013, 2015). In order to delineate a panel of biomarkers characteristic of fluoxetine response, we isolated PBMCs from the “most” affected CORT/Flx-R and CORT/Flx-NR animals.

### From β-Arrestin 1 Levels to a Differential Biological Signature of Fluoxetine Response in PBMC

Proteomic analysis has been described as a powerful tool for the identification of biomarkers (Bayes and Grant, 2009). Using high-resolution mass spectrometry, from 1245 proteins detected among the three conditions (CORT/V; CORT/Flx-R; CORT/Flx-NR), 305 were significantly and different expressed.

Surprisingly, among the proteins exhibiting variations compared to CORT/V group, Glyceraldehyde 3-phosphate dehydrogenase (GAPDH) showed a significant decreased in CORT/Flx-R and NR animals. A previous *in vitro* study demonstrated that antidepressants such as nortriptyline and escitalopram affect the expression of many housekeeping genes, including GAPDH (Sugden et al., 2010). From 10 housekeeping genes, GAPDH was the least stable protein detected in Mouse fibroblast L929 cells. Therefore, precautions should be taken in the use of certain housekeeping genes such as GAPDH, as its proteomic expression changes after antidepressant treatment.

Previously, we observed that β-arrestin 1 expression in PBMCs was restored to normal levels after chronic fluoxetine treatment in CORT-treated mice (Mendez-David et al., 2013). Here, we confirmed that β-arrestin 1 expression was significantly higher in CORT/Flx-R mice in comparison to CORT animals and a trend for its increase was observed between CORT/Flx-R *vs.* CORT/Flx-NR mice (*p* = 0.07). Although β-arrestin 1 expression in PBMCs has been proposed as a potential marker of antidepressant response in rodents (Mendez-David et al., 2013) and in depressed patients (Avissar et al., 2004; Matuzany-Ruban et al., 2005; Golan et al., 2013), MDD is more likely a multifactorial and polygenic disorder. Therefore, further investigation is needed to discover and validate other markers (Schmidt et al., 2011).

Hierarchical clustering of proteomic and PCA analyses showed that Flx-NR-animals are neighboring CORT-treated animals and are separate from CORT/Flx-R animals along the axis 1 of the PCA analysis with some overlap. While some animals seemed “mis-categorized” (CORT/Flx-R_28 and CORT/Flx-NR_76), these proteomic “outliers” displayed similar behavioral alterations compared to other animals within the same group. Fluoxetine effects on peripheral proteomic changes were mostly independent from antidepressant response as we observed a strong overlap across CORT/Flx-R and CORT/Flx-NR groups. 145 proteins were commonly observed with similar directional expression between responders and non-responders, even for proteins for which a statistically significant difference was observed between these groups. This is unsurprising as molecular peripheral markers of antidepressant response in clinical studies observed little variation in gene expression between responder and non-responder subjects for the same dosage of antidepressant (Pettai et al., 2016), thus confirming that the proteins differentially expressed in CORT/Flx-R and CORT/Flx-NR groups compared to the CORT-treated group are mostly witnesses of antidepressant exposure than of treatment response. Preclinical studies in the chronic mild stress model (CMS) also confirmed that overall central transcriptomic changes in the lateral habenula are more similar between responders and non-responders to escitalopram than between antidepressant-treated group compared to CMS or unstressed animals (Christensen et al., 2013). Unfortunately, the preclinical work that focus on genes/proteins differentially expressed (in peripheral tissue, cell lines, or within brain regions) between responders or non-responders did not evaluate the common effects of antidepressant treatment on gene expression (Christensen et al., 2011; Bisgaard et al., 2012; Park et al., 2016).

Here, we observed 100 proteins differentially expressed between CORT/Flx-R and CORT/Flx-NR among the 1245 detected proteins (8%), which is in a higher range compared to previous studies using transcriptomic/proteomic approaches in animal models of the disease (Christensen et al., 2011; Bisgaard et al., 2012; Park et al., 2016), or in human subjects (Mamdani et al., 2011; Guilloux et al., 2015; Pettai et al., 2016). However, methodological aspects may explain this difference between studies, as the number of groups, statistical methods and selection of responder/non-responder differ.

Interestingly, we show that the expression profile of these 100 proteins differs between CORT/Flx-R and CORT/Flx-NR (**Figure 5E**). Indeed, the effects caused by fluoxetine in CORT/Flx-NR generated a profile that strongly differed from the CORT-treated group. Importantly, the profile for CORT/Flx-R mice was closer to those found on the CORT/V group. This suggests that even if CORT/Flx-NR mice display a behavioral state similar to CORT/V-treated mice, their peripheral molecular state differs and might play a role in a “decanalization” process. This process has been proposed to explain the origin of complex diseases (Gibson, 2009; Gaiteri et al., 2010), and may be applicable to a lack of drug treatment-response. This group of proteins may also participate in an iatrogenic effect of fluoxetine.

Among these 100 proteins differentially expressed between CORT/Flx-R and CORT/Flx-NR mice, only 19 proteins were found with a differential direction of expression between CORT/Flx-R and CORT/Flx-NR or with a profile of expression in CORT/Flx-R that droves them away from the CORT/V-treated profile group. This list of proteins may be associated with an improvement in the emotional state of the animals and markers of antidepressant response. As most of them (12/19) showed similar direction of expression in CORT/Flx-R and NR, this also suggests that a threshold of expression needs to be exceeded in order to obtain an efficient antidepressant response.

Interestingly, an IPA analysis on these 19 biomarkers revealed that “*protein ubiquitination pathway*” and “*Interleukin 1 (IL-1) signaling*” are the top canonical pathways associated with this list (**Table 1**). The predominant role of ubiquitination is to target substrates for rapid degradation within the 26S proteasome, but also to regulate protein function by proteasome-independent processes. Previous clinical findings support the role of ubiquitination not only in the pathophysiology of MDD, but also in the mechanism of antidepressant-like activity (Golan et al., 2013). Ubiquitination of proteins such as β-arrestin 1 was demonstrated in PBMCs (Golan et al., 2013). In regards to the IL-1 signaling, many evidences support the role of cytokines and, more generally, inflammation in MDD and antidepressant drug treatment responsiveness [(Miller and Raison, 2016) for review]. In regards to the top disease and biological functions category, “*metabolic disease*” belongs to the top disease and disorders. A 6-month prospective, multicentric, a real-world treatment observational cohort study of 624 patients (METADAP) suggests that treating MDE with antidepressant drugs can induce or worsen a metabolic syndrome (Corruble et al., 2015). Actually, biomarkers of antidepressant response might be linked to these side-effects.

Importantly, 8 of the 19 proteins that exhibit differential direction of expression in Flx-responders *versus* Flx-non-responders have been associated with MDD or antidepressant response (RPN-2, HSPA9, NPTN, AP1B1, UQCRC2, RACK1, TOLLIP, and TLN2 (McHugh et al., 2008; Suzuki et al., 2010; Voloboueva and Giffard, 2011; Andrus et al., 2012; Porter et al., 2012; Beesley et al., 2014; Seguini et al., 2014; Waiskopf et al., 2014; O’Donovan et al., 2015; Xu et al., 2016; Bhattacharya et al., 2017; Hung et al., 2017). Finally, despite the few number of proteins used in this Ingenuity analysis, top networks included molecules belonging to “*Behavior*”, “*Nervous System Development and Function*”, which putatively suggest that we observe a peripheral signature of central differential response to fluoxetine treatment.

### Limitations of the Study

There is a great potential for biomarkers within the field of psychiatry, including diagnosis and/or prediction of treatment responses (Scarr et al., 2015). Whether or not the proteins involved in the fluoxetine response identified in PBMC are important for the pathophysiology, should also be studied. Moreover, at this stage, our study did not provide the means to directly assess brain markers and respectively, compare then with peripheral markers. Other limitation to address will be the sample size of the pilot study performed in an animal model of anxiety/depression, which was small, and an independent validation cohort was not available; hence, larger prospective pre-clinical studies are warranted.

The design of our study was to unravel the molecular and peripheral mechanisms associated with fluoxetine antidepressant response. Thus, no direct comparison between CORT/V-treated mice and controls was performed at the proteomic level, and the markers differentially expressed between responders and non-responders may not match with a peripheral signature of a behavioral state. Thus, this biosignature only reflects proteins associated with fluoxetine response/non-response. We should also consider that in our study, this peripheral proteomic biosignature of fluoxetine response was determined from the “most” affected mice from each group to increase group differences.

The biological validity of the inflammation and immune activation was not confirmed by other biological tests and is thus speculative at this time. Similarly, effects of fluoxetine on protein expression in naïve, unstressed animals, were not performed here. Thus, the possible iatrogenic effects of this antidepressant drug at the peripheral levels need to be confirmed.

Exploring protein expression profiles in homogeneous cell populations, such as PBMCs may provide a greater diagnostic power than whole blood signature. PBMCs include lymphocyte (T cells, B cells, and NK cells), monocyte and dendritic cells. In our study, the distribution of these cells in our experimental conditions has not been explored. We cannot rule out a decrease in blood PBMCs numbers. Indeed, leukocyte redistribution from the blood to other organs has been described (Dhabhar, 2006). Further studies should also consider an evaluation of PBMCs composition in the blood using flow cytometry. However, the extraction of more homogeneous cell populations, such as PBMC, which is often laborious and difficult to standardize, involves manipulation of the cells and may influence the expression profiles.

## Conclusion

In this study, we demonstrated that CORT model of anxiety/depression in mice allows the study of response/non-response to chronic fluoxetine treatment. We also provided evidence that even though CORT/Flx-R and CORT/Flx-NR groups share common proteins; a threshold of expression should be reached to categorize them as responders. These proteins represent a combination of markers associated both with the maintenance of a refractory state in these animals, while other may be associated with behavioral improvement. Whether these proteomic changes observed in PBMCs from CORT/Flx-R mice in the CORT model mirror biological changes or not in brain tissues, Further investigation is required.

## Author Contributions

IM-D, AG, DD: designed and performed research. IM-D, CB, VD, DD: performed research. IM-D, J-PG, DD drew figures. IM-D, CB, J-PG, RC, BF, EC analyzed the data. J-PG, RC, FB, EC: contributed to the preparation of the manuscript. IM-D, AG, J-PG, DD wrote the manuscript.

## Conflict of Interest Statement

The authors declare that the research was conducted in the absence of any commercial or financial relationships that could be construed as a potential conflict of interest.

## Footnotes

1 http://pappso.inra.fr

2 http://pappso.inra.fr/bioinfo/xtandempipeline/

3 http://pappso.inra.fr/bioinfo/masschroq/

4 www.ingenuity.com

## Supplementary Material

FIGURE S1 Change in emotionality *z*-score between behavioral sessions 1 and 2. **(A)** Normalization of data using *z*-score method was performed for each behavioral parameter in EPM, NSF, and ST after each behavioral session. Before fluoxetine treatment, emotionality score calculated after the first behavioral round did not differ significantly between groups corticosterone/vehicle (CORT/V), corticosterone/vehicle. **(B)** Normalization of data using *z*-score method was performed for each behavioral parameter in EPM, NSF, and ST after each behavioral session. Test *z*-values were then calculated by averaging individual *z*-scores, and averaged to obtain the emotionality score (emotionality scores 1 and 2: EM1 and EM2. Results are expressed as change in emotionality *z*-score obtained by subtracting the EM1 to EM2. Values plotted are mean ± SEM (*n* = 14, 12, 30, and 16 animals for vehicle/vehicle, corticosterone/vehicle, corticosterone/fluoxetine responder and corticosterone/fluoxetine non-responder per group, respectively). One-way ANOVA Fisher’s PLSD *post hoc* analysis (^∗^*p* < 0.05, ^∗∗^*p* < 0.01 *versus* vehicle/vehicle group; ^##^*p* < 0.01 *versus* corticosterone/vehicle group; ^§§^*p* < 0.01 *versus* corticosterone/fluoxetine’s responders group).

FIGURE S2 Emotionality score of the “most” affected mice of each groups used for proteomics analysis. Normalization of data using *z*-score method was performed for each behavioral parameter in EPM, NSF, and ST after the second session of behavior. Test *z*-values were then calculated by averaging individual *z*-scores, and averaged to obtain the emotionality score. Values plotted are mean ± SEM [*n* = 14, 5, 7, and 6 animals for vehicle/vehicle (VEH/V, open circle), corticosterone/vehicle (CORT/V, black dot), corticosterone/fluoxetine responder (CORT/Flx R, green dot), and corticosterone/fluoxetine non-responder (CORT/Flx NR, red dot) per group, respectively]. One-way ANOVA Fisher’s PLSD *post hoc* analysis ^∗∗^*p* < 0.01 *versus* vehicle/vehicle group; ^##^*p* < 0.01 *versus* corticosterone/vehicle group; ^§§^*p* < 0.01 *versus* corticosterone/fluoxetine’s responders group).

TABLE S1 Overall statistical results for behavioral studies.

TABLE S2 Protein identification by X!Tandem Pipeline across all samples.*Group ID:* Group which the protein belongs. All the proteins in a group have at least one peptide in common; *Sub-Group ID*: Sub-Group which the protein belongs. All the proteins in a sub-group are identified with the same valid peptides. The number of subgroups in which this peptide is present; *Protein ID:* A single reference to the protein in this grouping experiment (unique within a sample in individual mode); *Description:* Protein description as given by the identification engine. Protein description as it appears in the header of the fasta file; *log(E-Value):* Protein E-value expressed in log. Statistical value representing the number of times this protein would be identified randomly. Calculated as the product of unique peptide E-values in the sample; *Coverage:* Percentage of protein coverage; *MW:* Molecular weight of the protein expressed in KDa; *size:* The number of amino acid of the protein sequence; *Spectra:* Total number of scans (spectra) leading to the identification of this protein. The total number of spectra assigned by the identification engine to this protein; *PAI*: Protein Abundance Index: The PAI is an estimation of the abundance of the protein. PAI was defined as the number of sequenced peptides (fragmentation spectra assigned with significant score and as the top match to an individual identified protein) divided by the number of observable peptides per protein; *emPAI:* The emPAI is a PAI transformation such as: emP AI = 10P AI – 1.

TABLE S3 Protein spectral counting by X!Tandem Pipeline. *Group ID:* Group which the protein belongs. All the proteins in a group have at least one peptide in common; *Sub-Group ID*: Sub-Group which the protein belongs. All the proteins in a sub-group are identified with the same valid peptides. The number of subgroups in which this peptide is present; *Cort:* Corticosterone followed by animal ID; *Flx_NR:* Fluoxetine non-responder followed by animal ID; *Flx_R:* Fluoxetine responder followed by animal ID.

TABLE S4 Proteins significantly differentially expressed after one-way ANOVA. *Protein ID:* A single reference to the protein in this grouping experiment (unique within a sample in individual mode); *Cort:* Corticosterone; *Flx_NR:* Fluoxetine non-responder; *Flx_R:* Fluoxetine responder.
