# Supplementary figures and images for: Differential Peripheral Proteomic Biosignature of Fluoxetine Response in a Mouse Model of Anxiety/Depression
Source: Front Cell Neurosci. 2017 Aug 16;11:237. doi: 10.3389/fncel.2017.00237 (PMC5561647; doi:10.3389/fncel.2017.00237)

Supplementary Figure 1

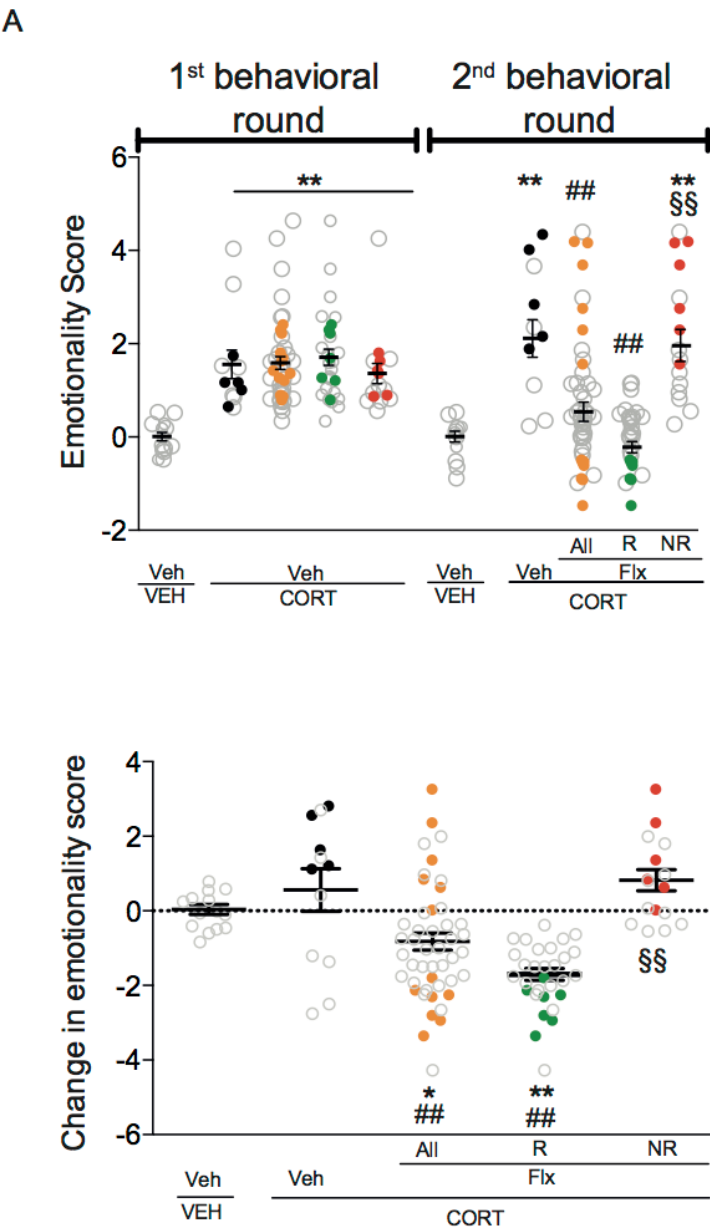

Supplementary Figure 2

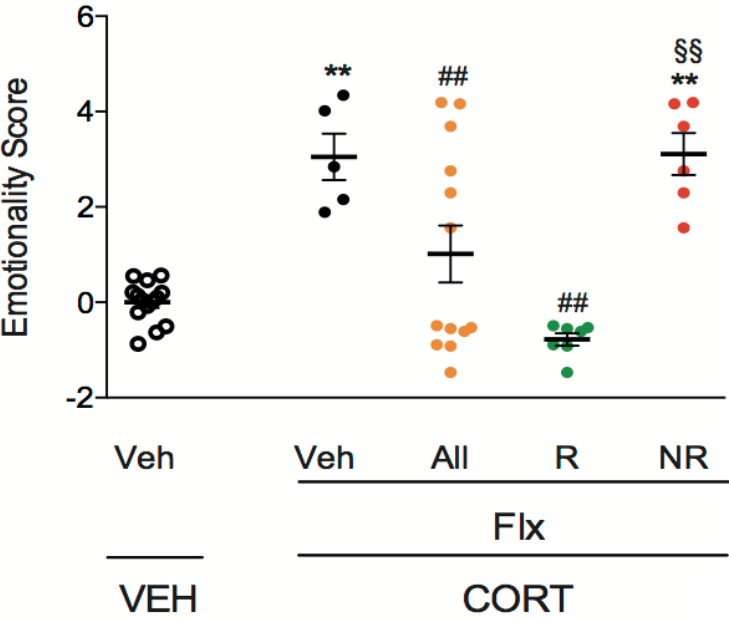

Supplement: FIGURE S1 — Change in emotionality z-score between behavioral sessions 1 and 2. (A) Normalization of data using z-score method was performed for each behavioral parameter in EPM, NSF, and ST after each behavioral session. Before fluoxetine treatment, emotionality score calculated after the first behavioral round did not differ significantly between groups corticosterone/vehicle (CORT/V), corticosterone/vehicle. (B) Normalization of data using z-score method was performed for each behavioral parameter in EPM, NSF, and ST after each behavioral session. Test z-values were then calculated by averaging individual z-scores, and averaged to obtain the emotionality score (emotionality scores 1 and 2: EM1 and EM2. Results are expressed as change in emotionality z-score obtained by subtracting the EM1 to EM2. Values plotted are mean ± SEM (n = 14, 12, 30, and 16 animals for vehicle/vehicle, corticosterone/vehicle, corticosterone/fluoxetine responder and corticosterone/fluoxetine non-responder per group, respectively). One-way ANOVA Fisher’s PLSD post hoc analysis (∗p < 0.05, ∗∗p < 0.01 versus vehicle/vehicle group; ##p < 0.01 versus corticosterone/vehicle group; §§p < 0.01 versus corticosterone/fluoxetine’s responders group). [file Image_1.PDF]
